# Development of a HIV-1 vaccine using an orally-administered, replication-competent adenovirus serotype 4 vector expressing Env clade C glycoprotein

**DOI:** 10.1186/1742-4690-9-S2-P35

**Published:** 2012-09-13

**Authors:** J Alexander, M Gurwith, J Mendy, L Vang, D Manayani, J Avanzini, B Guenther, P Farness, BF Haynes, H Liao, DC Montefiori, CC LaBranche, T Mayall

**Affiliations:** 1 PaxVax, San Diego, CA, USA; 2 Duke Human Vaccine Institute, Duke University School of Medicine, Durham, NC, USA; 3 Department of Surgery, Duke University Medical Center, Durham, NC, USA

## Background

Our hypothesis is that the replicating Ad4 vector approach, may be the best strategy for an effective HIV-1 vaccine due to advantages of demonstrated clinical safety and immunogenicity of both the Ad4 backbone and an Ad4 H5N1 vector influenza vaccine evaluated in Phase 1. Unlike other vectors, it can be bioengineered to express full-length HIV-1 Env gp160. More than 50% of global HIV-1 infections are caused by clade C viruses and therefore we initiated development of Ad4-Env160 vaccine using an Env clade C sequence obtained from CHAVI.

## Methods

The Ad4-Env viruses were evaluated for: 1) genetic stability; 2) Env protein expression by Western blot analysis; 3) cell-surface Env recognition by broadly neutralizing antibody (bnAb); and 4) immunogenicity in rabbits.

## Results

Genetically stable Ad4 recombinant viruses were generated which expressed the Env gp120, gp140, and gp160 proteins. A549 cells infected with Ad4-Env160 virus expressed cell-surface Env that was recognized by bnAb specific for MPER, CD4bs, V2-V3 loop sequences. Following immunization of rabbits, Env-specific binding antibodies were induced as measured by ELISA; 160>140>120.

## Conclusion

An Ad4 virus expressing full-length Env160 was generated and evaluated for genetic stability, protein expression, recognition by bnAb and immunogenicity. These results represent substantial progress towards defining a replicating Ad4 vector, recombinant protein vaccine prime/boost approach for HIV-1 that could eventually undergo clinical testing. Funding: NIH/NIAID SBIR 1R43AI091546-01; NIH/NIAID Contract No. HHSN266200400045C.

